# Characterization of Intracellular Structure Changes of *Microcystis* under Sonication Treatment by Polarized Light Scattering

**DOI:** 10.3390/bios11080279

**Published:** 2021-08-17

**Authors:** Jiajin Li, Chujun Zou, Ran Liao, Liang Peng, Hongjian Wang, Zhiming Guo, Hui Ma

**Affiliations:** 1 Institute for Ocean Engineering, Shenzhen International Graduate School, Tsinghua University, Shenzhen 518055, China; ljj18@mails.tsinghua.edu.cn (J.L.); whj20@mails.tsinghua.edu.cn (H.W.); gzm20@mails.tsinghua.edu.cn (Z.G.); 2 Department of Biomedical Engineering, Tsinghua University, Beijing 100084, China; 3 Guangdong Research Center of Polarization Imaging and Measurement Engineering Technology, Shenzhen International Graduate School, Tsinghua University, Shenzhen 518055, China; mahui@tsinghua.edu.cn; 4 Institute of Hydrobiology, Jinan University, Guangzhou 510632, China; 2018zcj@stu2018.jnu.edu.cn; 5 Department of Physics, Tsinghua University, Beijing 100084, China

**Keywords:** cyanobacterial blooms, *Microcystis*, gas vesicles, sonication, polarized scattered light, intracellular structure

## Abstract

Cyanobacterial bloom is one of the most urgent global environmental issues, which eventually could threaten human health and safety. Sonication treatment (ST) is a potential effective method to control cyanobacteria blooms in the field. Currently, the bottleneck of extensive application of ST is the difficulty to estimate the ST effect on the cyanobacterial cells and then determine suitable ST times in the field. In this study, cyanobacterial *Microcystis* samples sonicated at different times were first measured by a spectrophotometer to calculate the removal efficiency of *Microcystis* cells. Additionally, they were observed by TEM to reveal the intracellular structure changes of the cells. Then the samples were measured by an experimental setup based on polarized light scattering to measure the polarization parameters. Experimental results indicated that the polarization parameters can effectively characterize the intracellular structural changes of *Microcystis* cells with different ST times, which is quite consistent with the results for removal efficiency and TEM images. Further, the optimal ST time can be inferred by the polarization parameters. These results demonstrate that polarized light scattering can be a potentially powerful tool to explore suitable times for sonication treatment of cyanobacteria blooms.

## 1. Introduction

Nowadays, cyanobacterial blooms in eutrophic water bodies frequently cause deterioration of water quality [1], which can endanger the health of aquatic animals and plants as well as human beings [2], while at the same time increase the cost of water treatment [3]. Cyanobacterial blooms have become a serious and urgent environmental problem worldwide [4]. Hitherto, the main methods for controlling cyanobacterial blooms include shading, coagulation, filtration, algicides, and photolysis [5]. These treatment methods have played a certain role in algae removal. However, some are expensive or complex, and some can easily cause secondary pollution [6]. 

Recently, the environment-friendly sonication treatment (ST) has attracted increasing attention in research of the cyanobacterial removal process, because of its special selectivity to cyanobacterial cells, simple operation, low cost, mild reaction conditions, fast reaction speed, and no secondary pollution [7]. The widely acknowledged effects of ST on the growth inhibition of cyanobacterial cells are the collapse of gas vesicles, the disruption of membrane or cytoderm, and the interruption of photosynthetic activity [8]. 

Cyanobacterial cells can spontaneously adjust their buoyancy to migrate vertically in the water column by controlling the states of their gas vesicles, in order to be more adaptable to the external conditions. The cells can float to the surface of the water from the water column, and further, suddenly cause water blooms [9]. The main purpose of ST is to largely destroy the gas vesicles of cyanobacterial cells, causing the cells to lose buoyancy and sink to the bottom, so that the water blooms can be controlled [6]. For a fixed sonication power and frequency, we can generally get better growth inhibition of cyanobacterial cells with a longer ST time. However, with increasing ST time, saturation appears, and microcystin release may significantly increase [6,10]. For ST of cyanobacterial blooms, the high cost-performance means the optimal ST time needs to be determined [7]. Therefore, it is necessary for ST to monitor the intracellular structure changes of cyanobacterial cells in real-time, so as to estimate the treatment effect and determine the optimal ST time. However, it is still a big challenge for the research community to do this, which limits extensive application of ST in the field [7]. 

Previously, scientists generally used transmission electron microscopy (TEM) to observe the damage of cells caused by ST [6], and further, to determine the optimal ST time by observing the changes of the intracellular structures. It is effective and reliable, but time-consuming and expensive, and difficult to quickly measure the cells in situ. Moreover, chlorophyll-a assay and the fluorimeter are used commonly to evaluate effects of ST which are indirect and complex [6].

The biological characteristics of cyanobacteria interact with the light field through scattering and absorption processes [11,12]. The intracellular gas vesicles of cyanobacteria cells are significant bio-optical substructures [13,14], and potentially are one of the most important distinctive cellular structures influencing the optical properties [15]. 

Polarization is the fundamental property of light [16]. Polarized light scattering has been demonstrated as providing multidimensional parameters which are sensitive to the physical properties of particles, such as structure, size, and refractive index [17,18]. Recently, the development of polarized light scattering has attracted more and more attention from scholars, and the technique has been commonly used in atmospheric applications [19], bioscience [20], and water research [21], etc.

In previous works, we found that polarization parameters have the ability to characterize the collapse and recovery of gas vesicles of *Microcystis* cells after static pressure treatment or weak sonication treatment [17,21]. These works encouraged us to further explore the potential of polarized light scattering to break the bottleneck on determining the optimal ST time.

In this study, the *Microcystis* cell samples were sonicated at different times by a new self-developed sonication instrument with a fixed sonication power and frequency. First, we measured the samples using a spectrophotometer to calculate the removal efficiency of *Microcystis* cells. We also observed the cells by TEM and found that the intracellular structures of *Microcystis* cells can be destroyed to a different extent by various ST times, beyond the gas vesicles as in the previous papers [17,21]. Then the polarization parameters of the samples were measured by an experimental setup based on polarized light scattering. It was proved that polarization parameters can effectively characterize the intracellular structural changes of *Microcystis* cells under different ST times, which was quite consistent with the results of the removal efficiency and TEM. Further, the optimal ST time can be inferred by the relative standard deviation of the degree of polarization, which is a polarization parameter with explicit physical meaning. This study indicates that the polarized light scattering method may be a powerful tool to help sonication treatment control cyanobacterial *Microcystis* blooms in the field. 

## 2. Materials and Methods

### 2.1. Samples

The samples of cyanobacterial *Microcystis* were collected from *Microcystis* blooms in December 2020 from the surface of a pond in Zhuhai (22°8′ N, 113°16′ E). The cyanobacterial *Microcystis* is one of the most frequently reported cyanobacterial species for blooming and producing toxicity in natural water [22]. After the determination of the species by microscopy [22], we found that the *Microcystis* sample was dominated by *Microcystis aeruginosa*.

### 2.2. Sonication Treatment

**Figure 1:**
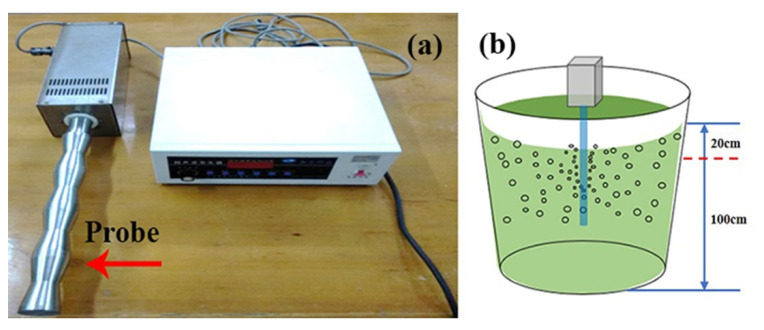
(**a**) The sonication instrument; (**b**) The schematic of sonication treatment.

In this study, a self-developed low-frequency sonication instrument was used to treat the cyanobacterial *Microcystis* samples, as shown in Figure 1a. The sonication instrument has a 50 mm diameter titanium probe, and the probe is put into 200 L *Microcystis* bloom water in the pail, as shown in Figure 1b. Previous studies have shown that a sonication parameter of frequency and intensity fixed at 20 kHz and 0.01 W/mL can enable effective removal efficiency of *Microcystis* cells [7,10]. Therefore, the bloom water was treated with the sonication instrument with a frequency of 20 kHz and a total power of 2000 W, for 0 s, 20 s, 40 s, 60 s, and 80 s, respectively. Meanwhile, the bloom water was stirred to ensure that the surface cells could be mixed into the range of the sonication probe. Finally, five *Microcystis* samples with different ST times were obtained.

The mentioned sonicated samples in the pail were allowed to stand for 2 h, after which 5 mL of the suspension was taken from 20 cm below the surface and the chlorophyll content measured. The chlorophyll pigments were extracted with 10 mL acetone (90%). The optical densities of the extracts were determined using a spectrophotometer, and further, the chlorophyll content could be computed [6]. 

The optimal ST time could be determined according to the removal efficiency of *Microcystis* cells in the bloom water. Herein, we can define *E* to quantify the removal efficiency of *Microcystis* cells in the bloom water [23], as shown in Equation (1).

(1) $$E=\left(1-\frac{C_{1}}{C_{2}}\right)*100,$$

where $C_{1}$ and $C_{2}$ are respectively the chlorophyll content of the bloom water after and before ST.

### 2.3. Experiment Setup of Polarized Light Scattering

**Figure 2:**
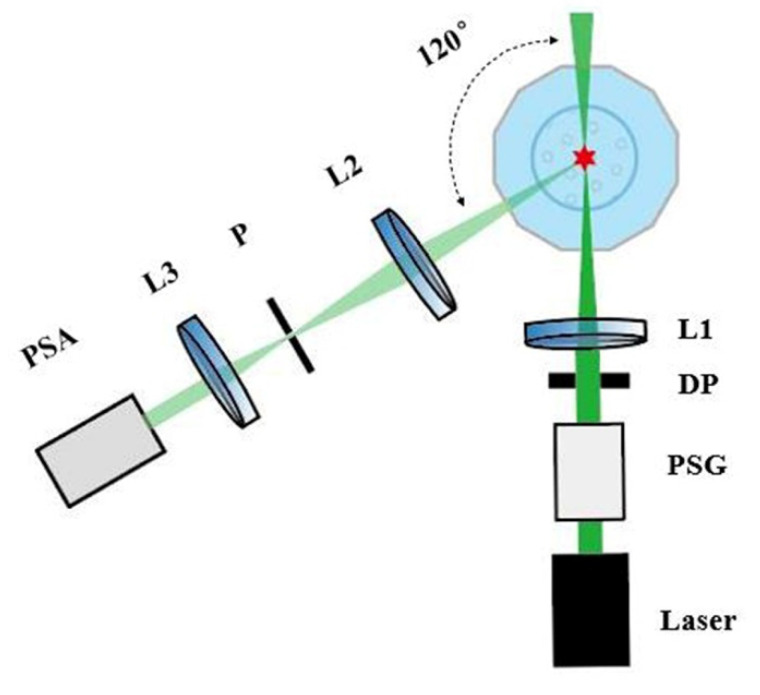
The experimental setup for polarization measurement.

An experimental setup was designed to measure the scattered polarization parameters of the suspended cyanobacterial *Microcystis* cells as individual particles [24], as shown in Figure 2. It consists of an illumination arm, a sample pool, and a detection arm. 

The sample pool consists of a glass dodecagon cuvette and a glass beaker. The glass beaker was placed at the center of the glass dodecagon cuvette which was filled with distilled water. The backscattered light at 120° was collected through the corresponding flat facet of the glass dodecagon cuvette. In our previous work, the 120° scattering angle was proved to characterize the microstructures of the particles [24]. The scattering of the bulk water at 120° is almost not sensitive to the size distribution of the particles [25]. Herein, we continued to use the 120° scattering angle in this work. The cyanobacterial cells were placed in the glass beaker and stirred by a magnetic stirrer at a speed of 200 rounds per minute.

In the illumination arm, the light source is a 200-mW laser at 532 nm wavelength. For the cyanobacterial *Microcystis*, there is little absorption of green light (532 nm) and the elastic scattering dominates the interaction of light with the cells, which allowed us to simply measure the polarization property of the cells. A polarization state generator (PSG) is used to change the polarization states of the illuminating light. In this work, we used 45° linearly polarized light as the illuminating light. The light beam passes through the diaphragm (DP) and then is focused by Lens 1 (L1) to a tiny focal spot whose size is less than 100 μm. In the detection arm, the light scattered by *Microcystis* cells is collected by Lens 2 (L2). The detection volume and the 100 μm diameter pinhole (P) is the object–image relationship via L2. P is carefully adjusted to ensure that the detection volume defined by L2 and P crosses the focal spot of L1, and the intersection volume is the scattering volume [16]. The scattered light passing through P is collimated by a Lens 3 (L3) and its polarization states can be detected by the polarization state analyzer (PSA) [24]. The measurement error of the polarization state of light is calibrated by using Thorlabs PAX1000VIS/M [24]. Herein, the concentration of *Microcystis* cells was controlled to less than 10^5^ particles per milliliter to ensure that there is only one cell in the scattering volume at most, which helps achieve the individual measurement of the cells. The signals consist of a series of temporal pulses in which each pulse corresponds to one particle and only the pulses whose signal-to-noise ratios are bigger than 5 are analyzed.

### 2.4. Analytical Methods

The Stokes vector S as defined in Equation (2), can be used to represent the polarization states of light.

(2) $$S=\left[\begin{array}{c} I\\ Q\\ U\\ V \end{array}\right]\;,$$

where I is the total light intensity, Q and U describe the residual polarization along the 0° and 45° directions respectively, and *V* describes the residual right circular polarization. After the polarization state of the scattered light is detected by PSA in Figure 2, the light signals are converted to voltages by photomultipliers and then digitalized by a data acquisition card and finally transmitted to a computer to calculate the scattered Stokes vectors. 

The degree of polarization (DOP) is a commonly used polarization parameter, which is defined as the proportion of the polarized light in the total light intensity, as shown in Equation (3). When the polarized light illuminates the suspended particles, the scatter may depolarize the illuminating light, and the DOP of the scattered light can be used to represent the degree of depolarization. In this case, the smaller the DOP of scattered light, the more is the depolarization caused by the scatter of particles [16].

(3) $$\mathrm{DOP}=\frac{\sqrt{Q^{2}+U^{2}+V^{2}}}{I}$$

Linear discriminant analysis (LDA) finds a linear combination of features of two classes of objects, in order to effectively characterize or distinguish between them [26]. The resulting combination can be used as a linear classifier. The linear combination maximizes the target function L as defined in Equation (4) to project the two data sets in a higher dimension space into a lower dimension space, and the two data sets are best separated after projection. Because the Stokes vector is multidimensional, we can use LDA to find the linear combination of the elements of the Stokes vector as a new polarization parameter to distinguish the samples.

(4) $$L=\frac{{\left(\mu_{1}-\mu_{2}\right)}^{2}}{\left(\delta_{1}^{2}+\delta_{2}^{2}\right)}\;$$

where $\mu_{1}$ and $\mu_{2}$ are the means of two data sets, while $\delta_{1}$ and $\delta_{2}$ are the standard deviations of two data sets after projection.

In this work, we normalized the elements of the scattered Stokes vector by the maximal intensity determined by the limit of the photomultipliers and the data acquisition card, such that all the elements of the Stokes vector were dimensionless and ranged from −1 to 1. Note that the elements of the Stokes vector and their derived parameters are all called polarization parameters. 

## 3. Results

### 3.1. The Effects of Different ST Times on the Microcystis Cells

**Figure 3:**
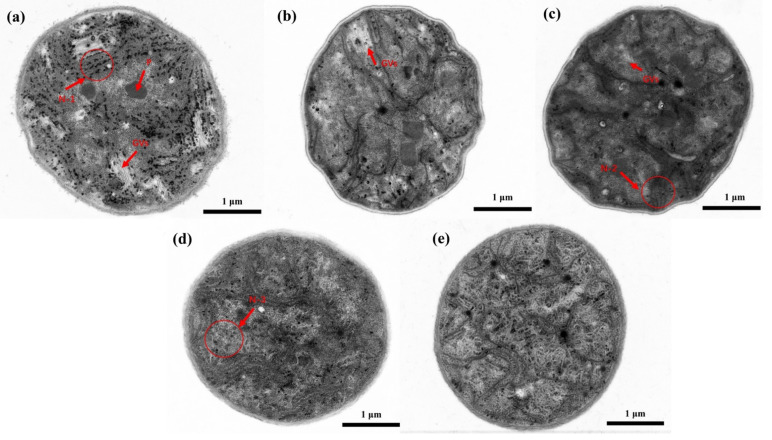
TEM images of *Microcystis* cells with different ST times, (**a**) 0 s; (**b**) 20 s; (**c**) 40 s; (**d**) 60 s; (**e**) 80 s. GVs, gas vesicles; C, cyanophycin granule; N-1, the attached phycobilisomes on the photosynthetic lamellae; N-2, the blurred and fractured photosynthetic lamellae; N-3, the converged intracellular structures.

Herein, TEM was used to observe the intracellular structure of the *Microcystis* cells after ST, as shown in Figure 3. According to TEM, it is noteworthy that different intracellular structures of *Microcystis* cells can be destroyed in sequence with the ST time. In Figure 3a, it is obvious that many gas vesicles (GVs) are distributed within the cell, and the cyanophycin granule (C) is clearly visible. Moreover, the phycobilisomes are attached to the outer surface of the photosynthetic lamellae in an orderly manner (N-1). In Figure 3b, the amount of the gas vesicles reduces sharply after 20 s ST, but we still can see a few gas vesicles in the cell. Besides, the photosynthetic lamellae are not damaged, but the amount of the phycobilisomes is reduced. In Figure 3c, it becomes hard to find the clear and complete gas vesicles, and the photosynthetic lamellae begin to become blurred and fractured (N-2). In Figure 3d, the gas vesicles disappear after 60 s ST. In addition, the intracellular structures begin to become converged, and the organelles start to disintegrate (N-3). In Figure 3e, the most remarkable feature is that the intracellular structures of the cell are completely destroyed and homogeneous after 80 s ST, but the cytoderm remains intact.

From 20 s to 40 s ST, the visible area of gas vesicles decreases sharply, but most of the other intracellular structures are still well kept. Particularly, after 40 s ST, the gas vesicles of the cells have a trend to disappear completely, and the photosynthetic lamellae appear to become blurred and fractured, which indicates the presence of a variety of cells in different states and diversity. Considering the different states of cells comprehensively, 40 s is the optimal ST time for the collapse of the gas vesicles. Any further ST will destroy the photosynthetic structure of the cells, and even all the intracellular structures, which affect the photosynthesis and nutrient storage of cells and accelerate the cells death. When a large number of cells die suddenly, the cells may rot and release more harmful toxins [6].

**Figure 4:**
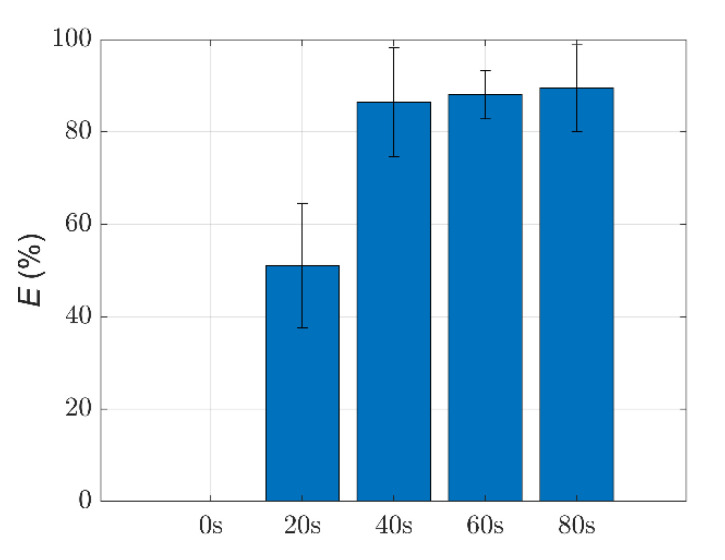
The removal efficiency *E* of *Microcystis* cells with different ST times.

Then, we calculated the removal efficiency *E* of *Microcystis* cells with different ST times, and the results are shown in Figure 4. The value of *E* can reach 51% after 20 s ST. Then, the value of *E* can increase 1.69 times after 40 s ST, compared with that of 20 s ST. Notably, a significant removal efficiency can be obtained during 0–40 s ST, and has no significant increase over 40–80 s ST. 

The buoyancy of *Microcystis* cells in water generally depends on the volume of the gas vesicles. If the gas vesicles of the cells collapse, the *Microcystis* cells will sink to the bottom of the water. Note that the samples are taken 20 cm below the surface of the bloom water. When the *Microcystis* cells start to sink to the bottom, the number of cells which stay in the water decreases, and *E* increases. When the gas vesicles in most *Microcystis* cells collapse, *E* will slow down and finally stop increasing. From Figure 4, one can find that the increase of *E* becomes slower after 40 s ST than that before 40 s ST; and this means that most of the gas vesicles have already collapsed inside the cells after 40 s ST, which is consistent with the TEM images. This implies that 40 s ST may be the optimal ST time to obtain a cost-effective treatment effect. 

### 3.2. The Polarization Measurement of Microcystis Cells with Different ST Times

**Figure 5:**
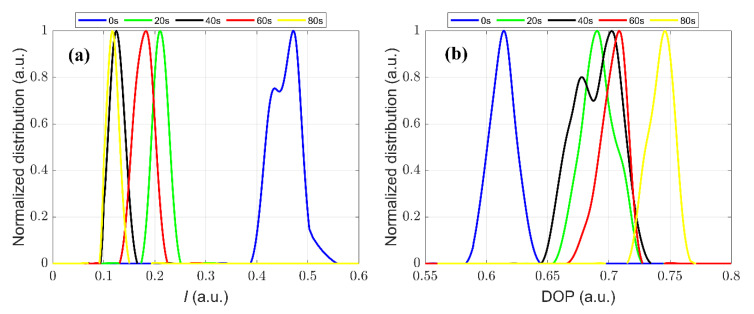
The distribution of (**a**) *I* (**b**) DOP for samples with different ST times.

The scattered Stokes vectors of *Microcystis* samples with different ST times were measured. For simplicity, we normalized the distributions of *I* and DOP after different ST times with their own maxima, which helped us to easily compare and analyze the shifts of peaks and the changes of the widths of the distributions. First, we observed the distribution of light intensity *I* of samples sonicated with different times, as shown in Figure 5a. Obviously, the change of *I* is not monotonic with the ST times. The scattered light intensity *I* reduces sharply after 20 s ST according to the 0 s ST and then the peak position of *I* shifts to the left after 40 s ST and next shifts to the right after 60 s ST. Particularly, the distributions of *I* after 40 s and 80 s ST are so close to each other that they cannot be effectively distinguished.

Then we investigated the distribution of DOP of samples with different ST times, as shown in Figure 5b. Similarly, DOP has a significant increase after 20 s ST compared to that after 0 s ST. After 40 s ST, the distribution of DOP becomes a double-peak structure and its main peak position moves to the right compared with that after 20 s ST. Then, the peak position of the DOP distribution moves towards the right after 60 s ST, and its distribution width becomes narrow. Next, DOP has a significant increase after 80 s ST. Notably, the distributions of DOP after 40 s and 80 s ST are so different that they can be effectively distinguished, which is quite different from the distribution of *I.*

**Figure 6:**
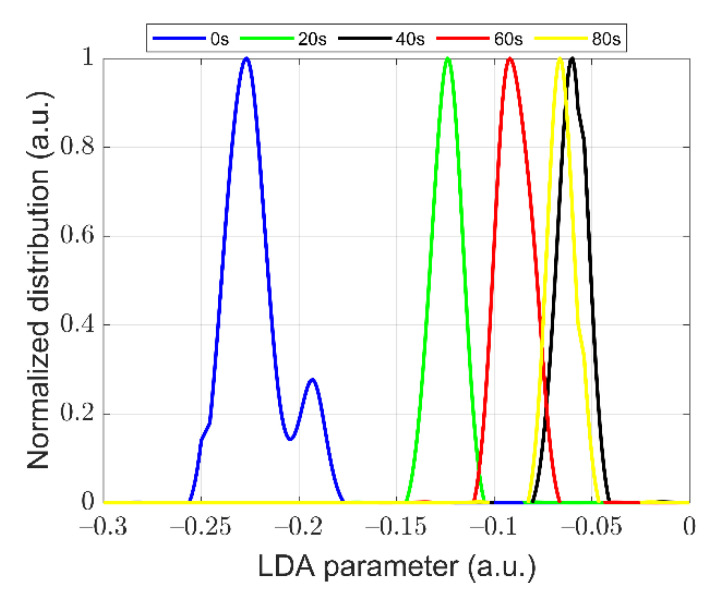
The distribution of LDA parameter of samples with different ST times.

We found that the *I* and DOP parameter are both not strong enough to recognize the samples after 20 s and 60 s ST. Thus, we tried to use LDA to look for a new polarization parameter with better recognition of them than *I* and DOP. The proposed LDA parameter is 0.02 × *Q* + 0.98 × *U* − 0.14 × *V*, which can more effectively discriminate the states of cells after 20 s and 60 s ST, as shown in Figure 6. The LDA parameter is used to extract and visually enhance the difference of the physical features between samples after 20 s ST and 60 s ST, and these physical differences are already included in the measured data.

**Figure 7:**
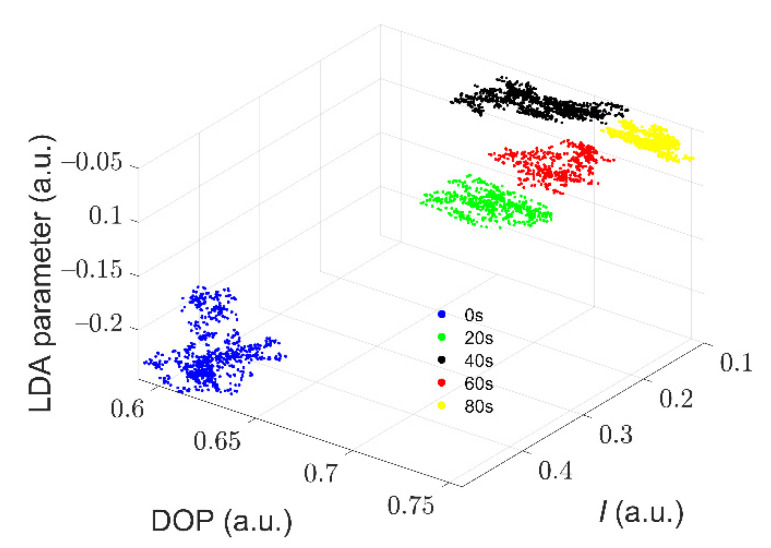
The distribution of the measured polarization parameters of *Microcystis* cells with different ST times.

From Figure 5 and Figure 6, it is hard to discriminate between all the distributions of the samples with different ST times using a single parameter, such as *I*, DOP, or the LDA parameter. Then, we tried to combine the parameters of *I*, DOP, and the LDA parameter to draw the data of cells in a three-dimensional space. As shown in Figure 7, it is apparent that the three parameters can effectively distinguish these *Microcystis* cells with different ST times. It demonstrates that the parameters of polarized light scattering can characterize the changes of the intracellular structure of *Microcystis* cells with different ST times.

### 3.3. Quantitative Characterization of Microcystis Cells with Different ST Times 

**Figure 8:**
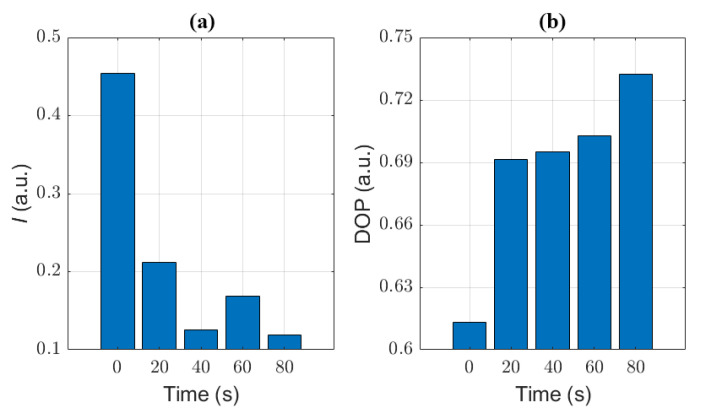
The mean of different samples with different ST times (**a**) *I*; (**b**) DOP.

In order to quantitatively characterize *Microcystis* cells after different ST times, we further calculated the mean values of light intensity *I* and DOP corresponding to *Microcystis* cells with different ST times, which is shown in Figure 8. Apparently, the changes of *I* are not monotonical, while the values of DOP change monotonically. It means that DOP may be better than *I* to characterize the changes of *Microcystis* cells with different ST times.

Notably, it can be found that in Figure 7 the distributions of samples are quite different. In order to facilitate the comparison, we defined the relative standard deviation (*D*_std_) to quantitatively characterize the dispersion of the distribution of DOP and *I* of different samples. The *D*_std_ is the ratio of the standard deviation of the distribution of parameters in the treated group to the standard deviation of the control group. If the *D*_std_ is greater than 1, it means that the distribution of parameters in the treated group is more disperse than that in the control group. Namely, the smaller the *D*_std_, the more concentrated is the distribution of the parameters of the treated group.

**Figure 9:**
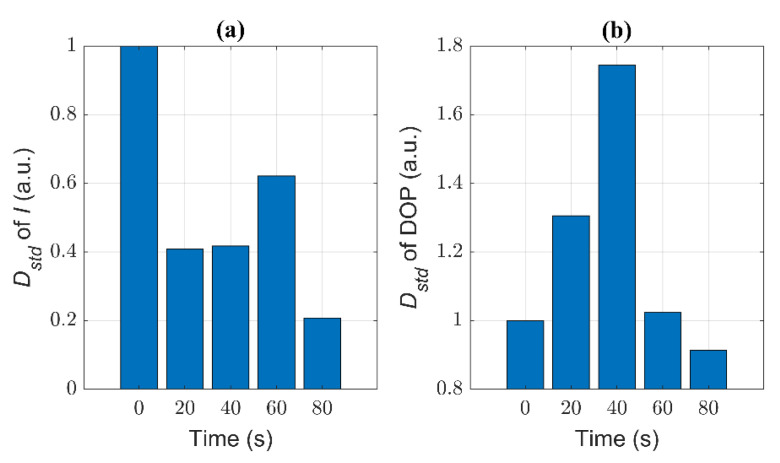
The relative standard deviation of samples with different ST times (**a**) *I*; (**b**) DOP.

In Figure 9a, we can see that the *D*_std_ of *I* reduces to 0.41 after 20 s and 40 s ST, but just reduces to 0.62 after 60 s ST, and finally reduces to 0.21 after 80 s ST, which means the *D*_std_ does not monotonically change with ST times. In general, the *D*_std_ of *I* becomes small after ST. 

In Figure 9b, with increasing ST time, the *D*_std_ of DOP first increases (the maximum is nearly 1.75), and then decreases. Particularly, it is obvious that the *D*_std_ of DOP reaches its maximum after 40 s ST. Recalling Figure 5b, the distribution of DOP has two main peaks after 40 s ST, and it can be inferred that there are two different main groups of components. Since DOP is more sensitive to the structural changes of particles [16], we can imagine that the presence of a variety of cells in different states leads to large variances. Moreover, when the cells are treated with 60 s ST, the intracellular structures become converged, and the *D*_std_ of DOP begins to reduce. After 80 s ST, it may be generally homogeneous inside the cells, the *D*_std_ of DOP is minimal. 

Recalling the analysis of TEM images and the changes of the removal efficiency of samples with different ST times, we can infer the optimal ST time (40 s) from the *D*_std_ of DOP.

## 4. Discussion

Experimental results in Figure 3, Figure 4, Figure 5, Figure 6, Figure 7, Figure 8 and Figure 9 indicate that we can characterize the intracellular structural changes of *Microcystis* cells with different ST times using polarization parameters. Further, we also can use the polarization parameters to infer the optimal ST time which is first determined by the removal efficiency and TEM images. 

There are several intracellular organelles such as gas vesicles, photosynthetic lamellae, and cyanophycin granules. The multiple scattering between these organelles depolarizes the illuminating polarized light, which leads to a lower DOP [18]. With the increasing ST time, the discrete organelles that act as scatterers inside the cells are gradually destroyed and the intracellular structures tend to become simple and convergent, and subsequently, the multiple scattering is reduced. Therefore, the values of DOP monotonically increase with ST time. 

However, for the untreated samples, the gas vesicles dominate the scattering and the *D*_std_ of DOP is small. After 20 s ST, the gas vesicles collapse dramatically and the inhomogeneity between the cells’ optical characteristics increases, so *D*_std_ of DOP increases quickly. After 40 s ST, the gas vesicles drop to a very low level and at the same time the discrete organelles tend to disorder, so the inhomogeneity between cells reaches a high level and the *D*_std_ of DOP reaches its maximal value. After 60 s ST, the gas vesicles almost disappear and the individual organelles start to disintegrate and the cells tend to show some similarity, which leads to the reduction of *D*_std_ of DOP. Finally, after 80 s ST, most organelles are destroyed, the whole cells appear uniform, and the intracellular difference between the cells is quite small, so the *D*_std_ of DOP is relatively the smallest. 

Some hints on the distributions of DOP with different ST times deserve paying attention to, as shown in Figure 5b. The distribution of the untreated sample is concentrated around the low mean value due to the dominant scattering of the gas vesicles. However, the 20 s ST lets the distribution shift to the right and becomes broad, and especially, there is a small secondary model located around 0.71. The 40 s ST directly splits the distribution of the cells into two models, and the main model closes to the former secondary model. From Figure 3c, we can see that the individual organelles are still clear but they are disordered, so it is acceptable that there are two compositions of cells in the sample which have two kinds of optical polarization properties. After 60 s ST, the secondary model of the distribution after 20 s ST grows up to the main part, which means that it is an inherent composition of cells after ST, and by comparison the others vanish until 60 s ST. Finally, after 80 s ST, the intracellular structures of all cells are heavily destroyed and the cells stay at the state with the DOP distribution concentrated around a high mean value, but the inherent composition still exists even though it is only a very small proportion. Generally speaking, the dominant composition of the cells after ST depends on the ST times. Particularly, 40 s ST can present all compositions, and from this point the inherent composition starts to be in the majority. 

From Figure 8, it can be seen that DOP can be a better parameter than the intensity *I* to characterize the intracellular structure of *Microcystis* cells with different ST times. However, the intensity is affected in a complex way by the size, structure, and refractive index of the cells and their intracellular organelle, so the intensity changes non-monotonically as the ST time. Because the gas vesicles can strongly scatter the light, the intensity *I* dramatically decreases after 20 s ST because of the collapse of most gas vesicles, and still further decreases after 40 s ST. Then, the other organelles may change but have weak scattering, which leads to the situation in which *I* fluctuates at low levels. Since the organelles begin to disintegrate after 60 s ST, the scattered intensity is subjected to the residual numbers of the organelles. This leads to the relatively wider variation range of the intensity *I*, and then larger *D*_std_ than those of 40 s and 80 s ST.

**Figure 10:**
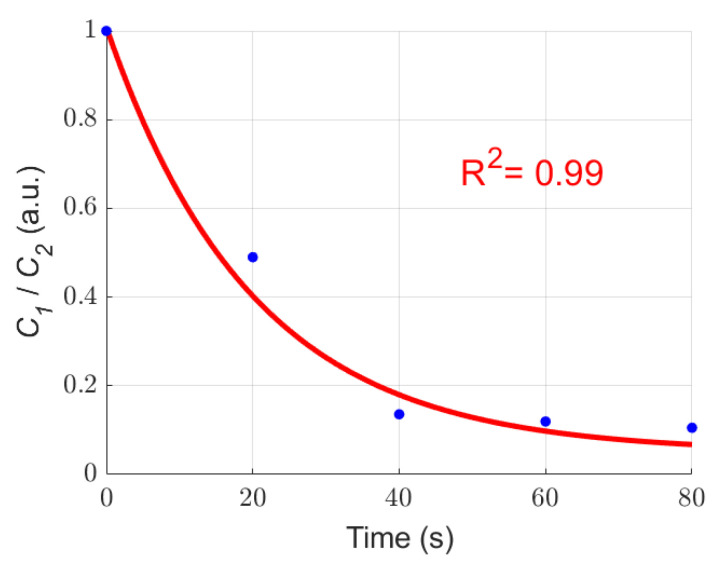
The exponential fitting curve of *C*_1_/*C*_2_ with different ST times.

To further study the applicability of our method, we calculated the chlorophyll ratio in Equation (1), *C*_1_/*C*_2_, from the measured removal efficiency, and used this ratio to represent the change of chlorophyll in the cells after ST. Then we found that the curve of the chlorophyll ratio is well fitted by the exponential relationship as shown in Figure 10 while the determination coefficient *R*^2^ is 0.99. Researchers have found a strong correlation between the chlorophyll content and the optical density [27]; and meanwhile, the optical density has an exponential relationship with the ST times [28]. This means that the experiments with different ST times in this work accord well with the natural rules revealed in previous literature. Even though our experiments were conducted only with several ST times, they stand on a very solid basis which founded by former researchers. Thus, the presented experiment design is effective, and our method was proven to be suitable for the characterization of ST on *Microcystis* cells.

**Figure 11:**
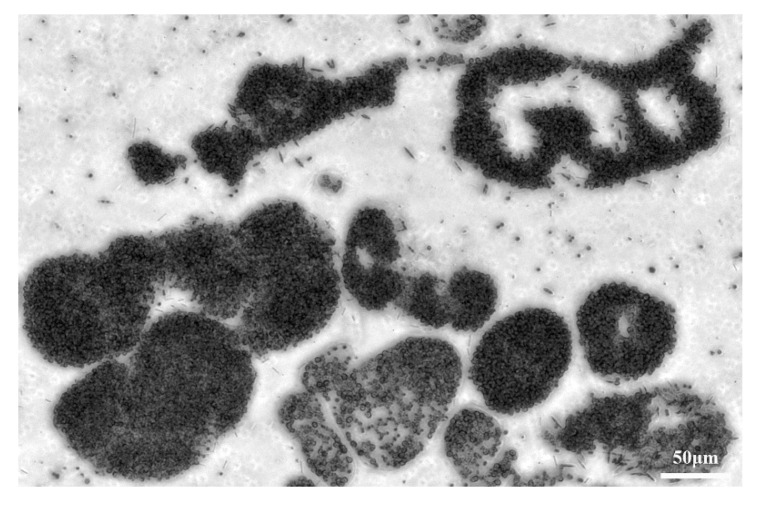
The micrograph of *Microcystis* cells.

The micrographs of untreated *Microcystis* samples are taken, and one of the micrographs is shown in Figure 11. We can find that the sample is dominated by *Microcystis aeruginosa*, but there are still many other *Microcystis* species with a different morphology [22]. Since for each sample, we measured more than 3000 individual cells, aggregations, or colonies, the interspecific and intraspecific differences of the particles with the aspect of size, shape, and morphology, were considered and included in the width of the polarization parameter’s distribution. Hence, the polarization parameters which can characterize the changes of samples under different ST, have hardly been influenced by the different kinds of *Microcystis* cells or colonic size and shape. These polarization parameters may be dominated by the intracellular structures in *Microcystis* cells, such as gas vesicles, photosynthetic lamellae, and cyanophycin granule.

In this study, we focused on the urgent demand to find a method to determine the optimal ST times when controlling cyanobacteria blooms in the field. The gas vesicles in the *Microcystis* cells and their buoyancy may recover after ST and then the cells will float to the surface of the water again, if the environmental factors such as temperature, nutrient, and illumination intensity, are suitable. The study on the relationship of different ST and the recovery of buoyancy of *Microcystis* cells is another interesting topic and should be conducted using the real-time polarized light scattering method in the future.

## 5. Conclusions

Cyanobacterial blooms are a globally emergent environmental issue, and sonication treatment has potential to effectively control the cyanobacterial blooms. In this paper, we presented a method based on polarized light scattering to characterize the intracellular structural changes of *Microcystis* cells after ST. An experimental setup was used to measure the scattered Stokes vectors of the suspended cells after different ST times. The results indicated that the polarization parameters based on the scattered Stokes vectors can characterize different *Microcystis* cells with different ST times, which is consistent with data for the removal efficiency and TEM images. Moreover, the relative standard variation of DOP can reveal the critical point at which the gas vesicles almost all collapse and the photosynthetic structures begin to be seriously damaged. Thus, the relative standard variation of DOP could be an indicator of the optimal ST time for cost-efficient control of cyanobacterial blooming. In summary, the proposed polarized light scattering method was demonstrated to be a powerful tool in characterizing the intracellular structural changes of *Microcystis* cells during sonication treatments.

## Data Availability

Not applicable.
